# Author Correction: Self-organization of an inhomogeneous memristive hardware for sequence learning

**DOI:** 10.1038/s41467-022-34322-5

**Published:** 2022-10-31

**Authors:** Melika Payvand, Filippo Moro, Kumiko Nomura, Thomas Dalgaty, Elisa Vianello, Yoshifumi Nishi, Giacomo Indiveri

**Affiliations:** 1 Institute for Neuroinformatics, University of Zurich and ETH Zurich, Zurich, Switzerland; 2 Université Grenoble Alpes, CEA, Leti, F-38000 Grenoble, France; 3 Corporate Research & Development Center, Toshiba Corporation, Kawasaki, Japan

**Keywords:** Electrical and electronic engineering, Information technology

Correction to: *Nature Communications* 10.1038/s41467-022-33476-6, published online 02 October 2022

The original version of this Article contained an error in the Acknowledgments, which incorrectly read ‘SNSF grant SMALL (20CH21_18699)’. The correct version states ‘SNSF grant SMALL (20CH21_186999)’ in place of ‘SNSF grant SMALL (20CH21_18699)’. This has been corrected in both the PDF and HTML.

