# Supplementary material for: Self-organization of an inhomogeneous memristive hardware for sequence learning
Source: Nat Commun. 2022 Oct 2;13:5793. doi: 10.1038/s41467-022-33476-6 (PMC9527242; doi:10.1038/s41467-022-33476-6)
Supplement: Supplementary file 1 — Supplementary Information [file 41467_2022_33476_MOESM1_ESM.pdf]

Supplemental Materials:
Neuromorphic memory Mosaic: re-configurable in-memory small-world graphs

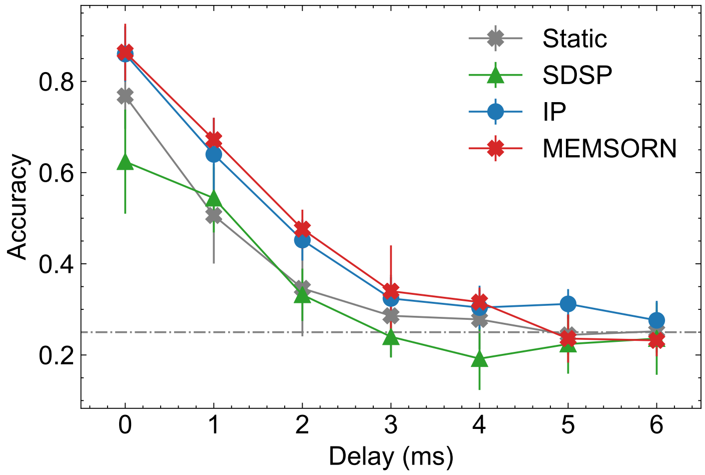

Figure S1: MEMSORN results on the RANDx4 task. Each input letter is randomly selected and presented to the reservoir for an interval of time approximately equivalent to the time constant of the neurons and synapse circuits. The formation of memory is thus solely attributed to the formation of a dynamical state mediated by the plasticity mechanisms. SDSP helps the reservoir to separate the response of the different inputs but has a negative impact on memory formation. IP makes the reservoir response more homogeneous across all neurons and improves the memory capacity. The combination of SDSP and IP allows for improving the performance, as demonstrated in earlier work [1].

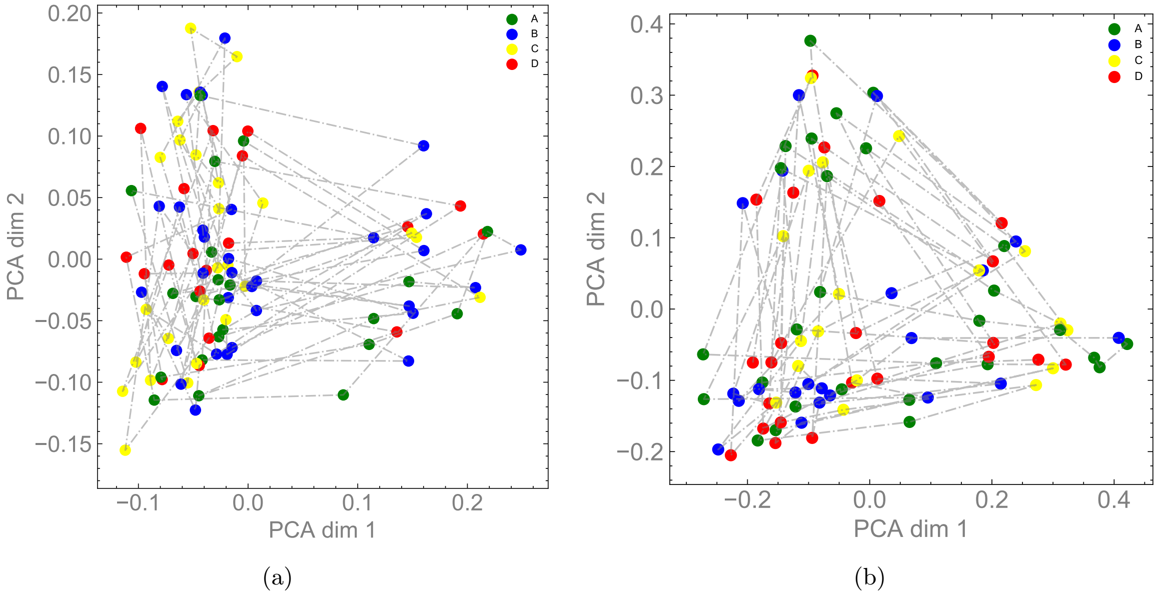

Figure S2: RANDx4 task: Principle Component Analysis (PCA) on the activity of the static network (a) and MEMSORN (b). The PCA on the MEMSORN shows a more cyclic trajectory than the static network. The cyclic nature helps to keep the information in the system in the form of short-term memory. The PCA justifies the larger accuracy of the MEMSORN.

Supplementary References

[1] Hazem Toutounji and Gordon Pipa. Spatiotemporal computations of an excitable and plastic brain: neuronal plasticity leads to noise-robust and noise-constructive computations. *PLoS computational biology*, 10(3):e1003512, 2014.
